# Holistic Processing of Words Modulated by Reading Experience

**DOI:** 10.1371/journal.pone.0020753

**Published:** 2011-06-16

**Authors:** Alan C.-N. Wong, Cindy M. Bukach, Crystal Yuen, Lizhuang Yang, Shirley Leung, Emma Greenspon

**Affiliations:** 1 Department of Psychology, The Chinese University of Hong Kong, Shatin, New Territories, Hong Kong; 2 Department of Psychology, University of Richmond, Richmond, Virginia, United States of America; University of Leuven, Belgium

## Abstract

Perceptual expertise has been studied intensively with faces and object categories involving detailed individuation. A common finding is that experience in fulfilling the task demand of fine, subordinate-level discrimination between highly similar instances is associated with the development of holistic processing. This study examines whether holistic processing is also engaged by expert word recognition, which is thought to involve coarser, basic-level processing that is more part-based. We adopted a paradigm widely used for faces – the composite task, and found clear evidence of holistic processing for English words. A second experiment further showed that holistic processing for words was sensitive to the amount of experience with the language concerned (native vs. second-language readers) and with the specific stimuli (words vs. pseudowords). The adoption of a paradigm from the face perception literature to the study of expert word perception is important for further comparison between perceptual expertise with words and face-like expertise.

## Introduction

Recent years have seen a surge of interest in the study of perceptual expertise for different object categories, such as faces [1], [2], cars [3], [4], fingerprints [5], music notes [6], and novel computer-generated objects [7], [8]. What is common among these categories is that expertise in a particular domain is often asfsociated with holistic processing, defined most commonly as the obligatory attention to all parts of an object [9], [10], or the emphasis on detailed spatial relationships between parts [11]. For example, holistic processing with cars was found for car experts only but not novices [3]. Indeed, holistic processing can be highly specific to subclasses within a domain, as demonstrated by the finding that modern car experts show holistic processing of modern but not antique cars [4] (but see [12] for a different interpretation). Moreover, holistic processing seems to occur only as a result of the right kind of experience, i.e., fine, subordinate-level discrimination among highly similar objects, but not coarser, basic-level classification [7], [13]. Therefore, holistic processing is thought to develop as an optimal strategy to fulfill the demand of fine discrimination, and can occur for objects other than faces as long as the same recognition demand is involved [14]. Here, we ask whether a similar type of holistic processing develops for expert word reading.

In contrast to expert face and object perception, the relationship between expert word perception and holistic processing has been more controversial, with some evidence suggesting that word reading is primarily part-based [15], [16], [17], other evidence suggesting that it is primarily holistic [18], [19], [20], [21], [22], [23], [24], [25], [26], [27], and some that it relies on the interplay of both holistic and part-based processing [28].

On the one hand, different behavioral and neural markers [29], [30], [31] seem to suggest that perception of text relies on a different kind of perceptual expertise. Whereas the above-mentioned “subordinate-level expertise” typically involves individuation of highly similar exemplars with the same general structure, words and letters require only basic-level recognition [32], [33]. Words differ from each other in terms of their general structure (e.g., number, identity, and order of letters), and detailed spatial relationships among letters are not informative of word identity. Part-based processing could therefore be more efficient for word perception than a holistic processing strategy. This idea is consistent with Farah et al.' s classic framework [17], [34] characterizing perception of different object categories as a continuum with part-based processing of words at one extreme and holistic processing of faces (or more recently, categories for which subordinate-level expertise is acquired) at the other. Other common objects lie somewhere in the middle.

Several pieces of evidence suggest that word perception occurs mainly in a part-based manner. Farah *et al.* [17] showed that words are masked equally effectively by another word and by the same word mask with the letters shuffled, whereas faces are only effectively masked by another face but not when the parts of the face mask are scrambled. Another study examined letter and word recognition efficiency when presented in background noise [16]. This study found that recognition efficiency was inversely proportional to word length, and that accuracy never exceeded that predicted by a letter-by-letter computational model. Martelli et al. [15] addressed this question from a different perspective using the phenomenon of crowding. They showed that in peripheral vision there needs to be enough spacing between letters of a word so that each letter can be isolated from the others for the word to be recognizable. In other words, word recognition requires the isolation of parts and thus is part-based. One could question, however, whether perception of impoverished situations (by masking, noise introduction, or peripheral presentation) is representative of normal word perception processes [35].

On the other hand, one could argue that expert word perception involves at least certain aspects of holistic processing. Regularities in words (i.e., orthography) can result in the formation of chunks, according to statistical learning research [27]. In fact, there is the well-known word superiority effect [25], [26], in which letters are better recognized in the context of a word than in isolation. These data suggest that whole word representations exist and can affect recognition at the letter or feature level [23], [24]. Other studies have shown successful word recognition even when perception of constituent letters is seriously impaired [21], [22]. The importance of global word shape has also been shown in normal reading situations [18], [19], [20].

Finally, it has also been proposed that word reading relies on both part-based and holistic processes, depending on the context [28]. According to this framework, expert holistic processing of words is subserved by a ventral occipito-temporal pathway that is organized hierarchically from posterior to anterior, with neurons in more anterior regions (visual word form area) responsible for holistic processing (parallel encoding). Part-based processing is subserved by the dorsal pathway and is responsible for serial attention to letters whenever context is not optimal for whole word reading. Children under the age of 10 are thought to rely more heavily on the dorsal route if they have not developed a sufficient level of expertise.

The current study attempts to bridge the study of holistic processing between face and word perception (see also Farah et al. [17], Ge *et al.* [36], and Hsiao & Cottrell [37]). We focused on one aspect of holistic processing: the obligatory processing of all parts of a stimulus [9]. We adopted a composite matching task typically used to demonstrate holistic face processing [10], with a complete-design modification that allows a bias-free estimate [2], [38], [39]. This task has been frequently used to demonstrate holistic processing for faces and other objects [3], [7], [40], thus it would be informative to examine how words would fare in the same task. In fact this task has been used previously for studying holistic processing of single Roman letters [41] and Chinese characters [37]. Interestingly, studies using Chinese characters have shown that expertise is associated with reduced reliance on detailed spatial relations between parts [36], and with a better ability to selectively attend to part of a character [37]. The generalizability of these findings to alphabetic writing systems remains to be seen.

**Figure 1:**
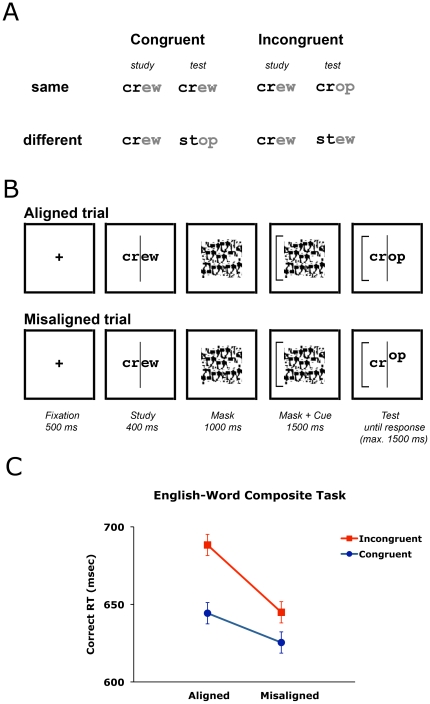
Details of Experiment 1. (A) An example set of English words and their assignment to different conditions. In this example trial, matching of the left part is required, and the black parts are to be attended to while the grey parts are to be ignored (for illustration only; in actual experiment both parts are black). (B) The trial sequence. (C) Mean response times. Error bars represent 95% confidence interval for the congruency factor.

The composite task also has the benefit of giving a stringent test of holistic processing: the task demands matching of only part of the stimuli and any interference from the irrelevant part would indicate automatic and compulsory processing of information from all parts of a stimulus. In Experiment 1 we applied the composite task to examine the holistic processing of words (Figure 1). On each trial, participants were cued to match either the left or right halves of two sequentially presented four-letter words, while ignoring the noncued half. Half of the trials were congruent (both left and right halves matched or mismatched) and half of the trials were incongruent (one half of the word matched and the other half mismatched). Better performance in the congruent than incongruent condition would indicate interference from the irrelevant part, hence imperfect selective attention to the target part of the stimulus. Interference may also be reduced when the configuration of parts is disrupted at test according to what has been found for faces. The two halves of the words were therefore vertically misaligned at test on half of the trials. Following the face literature, holistic processing of words is defined in two ways: (i) the better performance for congruent than incongruent trials in the aligned condition; and (ii) the larger congruency effect in the aligned than misaligned condition.

One could argue that any holistic processing may merely reflect a general bias towards global processing for all object categories [42], [43]. If this is the case, then we should not expect the holistic processing found to be sensitive to experience with words. Therefore, in Experiment 2, we investigated whether the effects found in Experiment 1 are driven by experience. We recruited native English readers and Chinese readers who learned English as their second language (ESL). In a sense they are both experts with the English words, but with different levels of expertise. The native English readers were expected to show larger holistic processing due to their more extensive experience with English words and higher proficiency with the English language. Apart from differences among groups, experience also varies among different items. For example, holistic processing has been shown to be larger for familiar than unfamiliar faces [44], [45], [46]. We therefore also probed the effect of experience within each group, by introducing three different types of stimuli: (a) words with a high written frequency, (b) words with a low written frequency, and (c) pseudowords (pronounceable nonwords). It should be noted that while the three types of stimuli differ in the amount of experience a native reader would have (largest for high-frequency words and smallest for pseudowords), no assumptions were made concerning the role that specific types of experience (visual, linguistic, or both) would contribute to differences in holistic processing if observed. If holistic processing depends on our experience with the individual stimuli, then we should expect holistic processing to be the largest for high-frequency words and the smallest for pseudowords. Such modulation by word type should be apparent for the native English readers due to their extensive experience with English words. For ESL readers, though they were fluent in English reading, the majority of their reading experience involved Chinese rather than English. Therefore their experience with English was much less than that for native readers, and any differences in holistic processing among word types may only emerge after more extensive experience with the language. We thus expect the different word types should have a smaller or no effect on holistic processing for ESL readers.

## Methods

### Experiment 1

#### Ethics Statement

The procedures have been approved by the Institutional Review Board of the University of Richmond. Informed consent was obtained in written form from all participants.

#### Participant

Seventeen native English readers (6 males, mean age  = 19.9) were recruited at the University of Richmond. They all had normal or corrected vision, and were given monetary compensation for their participation.

#### Material

Ten sets of four-letter words were used to create the stimuli for different conditions (see Table S1 in the Supporting Information). Words had a mean frequency of 78.9 per million (SD = 118.4 per million) according to the Kucera-Francis written frequency measure [47]. Each set contained four words such that the left and right halves could be interchanged to create the four test conditions (see Figure 1A). Within each set, each word appeared as the study stimulus with the same frequency; each word also appeared as the test stimulus equally often in the four different conditions. Each word spanned 3.4° of visual angle vertically and 9.5° horizontally with a 60-cm viewing distance. Left and right halves of words were separated by a vertical line. Stimuli were presented on a 17-inch Mac computer using MATLAB™ (MathWorks, Natick MA) and Psychophysics Toolbox [48].

#### Procedure

Participants viewed a fixation cross for 500 ms and then the study stimulus for 400 ms (Figure 1B). A mask then appeared for 2500 ms, followed by the test stimulus. A cue on the left (or right) appeared at the last 1500 ms of the mask and remained on the screen during presentation of the test stimulus. The test stimulus remained on the screen until participants responded. Participants had to indicate by key press (“1” for “same” and “2” for “different”) if the left (or right) part of the study and test stimuli were the same within 1500 ms. No feedback was given. In half of the trials, the two parts of the test stimulus were misaligned by moving the noncued part vertically by about 1.7°.

There were 40 trials for each of the 16 conditions (left/right matching × alignment × congruency × same/different response), forming a total of 640 trials divided into 16 blocks. Each of the 40 words (10 sets each with 4 words) was presented in the study 16 times. The different types of trials were randomized except that half of the participants had all aligned trials followed by misaligned trials, while the other half finished all misaligned trials first. Participants completed 10 practice trials before the actual experimental trials began.

### Experiment 2

#### Ethics Statement

The procedures have been approved by the Survey and Behavioral Research Ethics Committee of the Chinese University of Hong Kong, and by the Institutional Review Board of the University of Richmond. Informed consent was obtained in written form from all participants.

#### Participants

Forty-six native English readers (16 males, mean age  =  19.6) were recruited at the University of Richmond. Fifty-one ESL readers (22 males, mean age  =  20.7) who all had Chinese as their mother tongue and learned English as a second language for more than 15 years were recruited at the Chinese University of Hong Kong. They all had normal or corrected vision, and were given monetary compensation for their participation.

#### Material

Twelve sets of words were used to create the stimuli (see Table S2 in the Supporting Information). Four of the sets consisted of high-frequency words (mean = 284.3, SD = 463.8) and four of low-frequency words (mean = 3.13, SD = 1.63) according to the Kucera-Francis written frequency measure [47]. The remaining four sets were pseudowords (i.e., pronounceable nonwords). Word types were matched for number of ascenders and descenders. The size and presentation conditions of the stimuli were identical as those in Experiment 1.

#### Procedure

Trial sequence was similar to that in Experiment 1, except that the mask in each trial was shortened to 800 ms (with the cue appearing at the last 300 ms of the mask) to accommodate the greater number of trials. For each of the 12 conditions (word type × alignment × congruency), there were 64 trials, with half being left-matching and half right-matching, and also half same and half different trials. There were thus a total of 768 trials divided into 24 blocks. Each of the 48 words (12 sets each with 4 words) was presented in the study 16 times. All types of trials were intermixed except that different types of words were shown in different blocks and the order of blocks were counterbalanced within and across participants. Twelve practice trials with feedback were introduced before the actual experimental trials. After the experiment, all participants (except for three native readers) wrote down the meaning for the words used in the experiment on a sheet of paper. Both groups of participants knew the meaning of a higher proportion of high- than low-frequency words [native readers: 0.969 vs. 0.779, *t*(42) = 10.57, p<.0001, *d* = 1.61; ESL readers: 0.953 vs. 0.264, *t*(50) = 54.84, p<.0001, *d* = 7.55].

## Results

### Experiment 1

Response times (RT) for correct trials and sensitivities (A') are shown in Figure 1C below and Table 1 respectively. A' is a non-parametric measure of sensitivity according to the signal detection theory without the assumption of normality or that of equal variances [49]. It is calculated as:
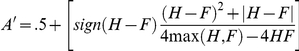
where *H* and *F* represent hit rate and false alarm rate respectively. We focus on reporting the statistics on RT, because (i) the overall sensitivity was very high (A' = .959); and (ii) sensitivity measures showed a similar result pattern as RT though were less sensitive to differences among conditions.

**Table 1:** Sensitivity (A') measures in Experiment 1.

Conditions		A'
Aligned	Congruent	0.968
	Incongruent	0.959
Misaligned	Congruent	0.968
	Incongruent	0.958

Responses were faster in the congruent than incongruent condition, and this difference was larger for aligned than misaligned trials, a pattern typically found for face perception. This was confirmed with a 2 (Congruency) × 2 (Alignment) analysis of variance (ANOVA) which showed a significant Congruency effect [*F*(1,16) = 29.46, *p*<.0001, *η*_p_^2^ = .648] and Alignment × Congruency interaction [*F*(1,16) = 4.28, *p* = .05, *η*_p_^2^ = .211]. There was no significant Alignment effect [*p*>.22]. Scheffé's tests (*p*<.05) showed significant difference between congruent and incongruent conditions for both aligned and misaligned trials.

### Experiment 2

**Figure 2:**
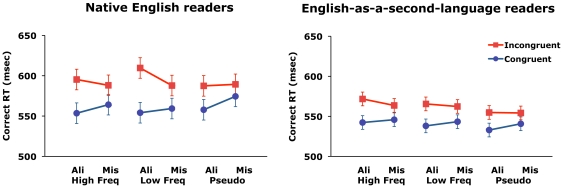
Mean response times in Experiment 2. Error bars represent 95% confidence interval for the congruency factor.

Response times (RT) for correct trials and sensitivities (A') are shown in Figure 2 and Table 2. Sensitivity was high overall (A' = .975) and showed similar though smaller differences among conditions compared with response times.

**Table 2:** Sensitivity (A') measures in Experiment 2.

Group	Type	Conditions		A'
Native English readers	High-frequency words	Aligned	Congruent	0.985
			Incongruent	0.966
		Misaligned	Congruent	0.984
			Incongruent	0.968
	Low-frequency words	Aligned	Congruent	0.981
			Incongruent	0.962
		Misaligned	Congruent	0.976
			Incongruent	0.969
	Pseudowords	Aligned	Congruent	0.978
			Incongruent	0.968
		Misaligned	Congruent	0.979
			Incongruent	0.967
Readers with English as the second language	High-frequency words	Aligned	Congruent	0.985
			Incongruent	0.963
		Misaligned	Congruent	0.985
			Incongruent	0.974
	Low-frequency words	Aligned	Congruent	0.979
			Incongruent	0.97
		Misaligned	Congruent	0.976
			Incongruent	0.975
	Pseudowords	Aligned	Congruent	0.986
			Incongruent	0.976
		Misaligned	Congruent	0.982
			Incongruent	0.973

Both groups of participants responded faster in the congruent than incongruent condition, and the difference was larger for aligned than misaligned trials. The congruency effect appeared larger for native English readers, and it also appeared to depend on word type for English readers but not for ESL readers. We first present results of the overall omnibus ANOVA, followed by the planned comparisons on RT that test our specific hypotheses concerning the effect of experience on holistic processing.

A Group × Word Type × Alignment × Congruency mixed factorial ANOVA conducted on the RT data showed larger holistic processing for native than ESL readers [Group × Alignment × Congruency: *F*(1,95) = 4.72, *p* = .032, *η*_p_^2^ = .047; Group × Congruency: *F*(1,95) = 6.32, *p* = .013, *η*_p_^2^ = .062]. There was also a three-way interaction between Group, Word Type and Congruency [*F*(2,190) = 3.33, *p* = .037, *η*_p_^2^ = .033]. To investigate these differences more thoroughly, we conducted separate 3-way analyses for the two groups. The native English readers showed an effect of Congruency [*F*(1,45) = 78.28, *p*<.0001, *η*_p_^2^ = .634] and an Alignment × Congruency interaction [*F*(1,45) = 25.52, *p*<.0001, *η*_p_^2^ = .361]. In addition, Word Type interacted with both Congruency [*F*(2,90) = 10.48, *p*<.0001, *η*_p_^2^ = .188] and Alignment [*F*(2,90) = 11.08, *p*<.0001, *η*_p_^2^ = .197]. No interaction between Word Type, Alignment, and Congruency was found [*p*>.29]. The ESL readers showed a significant effect of Congruency [*F*(1,50) = 76.32, *p*<.0001, *η*_p_^2^ = .604] and an Alignment × Congruency interaction [*F*(1,50) = 11.07, *p*<.0001, *η*_p_^2^ = .181]. No interaction was found between Word Type and any other variables [*p*s>.20].

We conducted planned comparisons to test our two hypotheses concerning the dependence of holistic processing on experience. Following past studies, holistic processing was defined in two ways: (i) as the congruency effect (incongruent RT – congruent RT) for aligned trials, indicating how much the observer is interfered by information in the irrelevant part [4], [17], [50], [51]; and (ii) as the effect of alignment on the congruency effect [(aligned_incongruent RT – aligned_congruent RT) – (misaligned_incongruent RT – misaligned_congruent RT)], indicating how much the interference from the irrelevant part depends on the intact configuration of parts [2], [4], [7], [52], [53]. The second measure, i.e., the interaction between alignment and congruency, has been found to be predictive of face recognition performance [2], and also especially sensitive to experience for non-face objects [7], [52].

Our first hypothesis was that holistic processing would be larger for native English readers than ESL readers. Planned comparison showed such a difference in the congruency effect [*t*(95) = 3.22, *p* = .001, *d* = .66] and also in the dependence of the congruency effect on alignment [*t*(95) = 2.17, *p* = .032, *d* = .45].

Our second hypothesis was that holistic processing would be modulated by word type, especially for native English readers. Planned comparisons showed that, for native readers, the congruency effect was smaller for pseudowords compared with high-frequency words [*t*(45) = 2.09, *p* = .041, *d* = .234] and with low-frequency words [*t*(45) = 3.92, *p*<.001, *d* = .506]. Interestingly the congruency effect was larger for low- than high- frequency words [*t*(45) = 2.304, *p* = .025, *d* = .339]. For the ESL readers, however, there was no significant difference in the congruency effect among the three word types [*p*s>.18]. There was also no significant difference in the effect of alignment on the congruency effect among the three word types for each group [*p*s>.13].

It is interesting that, despite over 15 years of English learning experience, the ESL readers showed less holistic processing for English words than native readers. Previous studies with ESL readers of a similar background have shown that they have a comparable level of expertise with the Roman alphabets as native English readers, in terms of recognition performance and neural activity level [31], [32]. It is thus an intriguing possibility that the smaller holistic processing for the ESL readers resulted from their lower visual experience with whole words or less consolidated linguistic knowledge.

Apart from the group difference, the native English readers also showed different degrees of holistic processing for different word types: while the larger holistic processing for words relative to pseudowords is expected, curiously the effects were the largest for the low-frequency words. Whereas the greater holistic processing for words than pseudowords can be attributed to greater experience with words than pseudowords, the greater holistic processing for low frequency than high frequency words is much more difficult to interpret. Due to the difficulty in forming the word sets for the composite task such that all combinations of bigrams resulted in legal words of a particular frequency range, a number of extraneous factors (e.g., bigram frequency, lexical neighborhood, concreteness, imageability, meaningfulness, age of acquisition, etc.) may also have differed between high and low frequency conditions in addition to word frequency. The contrast between words and pseudowords is therefore the more informative comparison and we believe should receive emphasis here.

## Discussion

In two experiments, we showed robust holistic processing for words: matching target parts of a word was interfered by the irrelevant parts, and such interference was reduced when the parts were misaligned. We also showed that holistic processing was sensitive to the amount of experience with the stimuli, in that it was larger for native than second-language readers, and larger for words than pseudowords in native readers. The relative contribution of perceptual and semantic experience on holistic processing is not within the scope of the current study, though these factors could be dissociated in a training paradigm that compares visual training alone with visual and semantic training of words in a novel writing system.

Our results seem opposite to what was found in Hsiao & Cottrell's study using the same composite paradigm [37], in which non-Chinese readers showed holistic processing for Chinese characters with well-aligned parts while Chinese readers did not. According to Hsiao and Cottrell, the non-Chinese readers had difficulty separating the components due to their lack of knowledge with Chinese characters. The novice situation in their study was rather different from what the ESL readers faced in our study, because (i) the left and right parts of each word are much easier to decompose (unlike the spatially joined top and bottom parts of each Chinese character used); and (ii) our ESL readers had much more experience with the English words than non-Chinese readers had with Chinese characters. Still this cannot explain why holistic processing was found for experts in our study but not in theirs. A possibility concerns the ceiling effect that may have masked the holistic processing for experts in Hsiao and Cottrell's study: While they used sensitivity (A') as their primary measure, in most conditions the Chinese readers performed almost perfectly (A'>.95). With the ceiling effect avoided, perhaps by using a sequential instead of a simultaneous matching task so that response times could also be analyzed, we expect that Chinese readers should also show obligatory attention to all parts of a character and thus holistic processing.

While the difference between native and ESL readers can be attributed to their differential experience with English words, an alternative possibility is that ESL readers showed less holistic processing because of the difference in native writing system. It could be that previous experience with the Chinese writing system was sufficient to render the Chinese readers part-based processors, such that lower holistic processing would be observed for both Chinese characters and English words. This seems unlikely, however, because previous studies showed that expertise effects are highly domain-specific. The most extreme example is that modern car experts showed holistic processing only for modern cars but not for antique cars [4]. Therefore it is quite a leap to assume that expertise with the Chinese writing system would cause reduced holistic processing readily generalizable to English words. Again, re-examining holistic processing for Chinese characters in experts and novices with the ceiling effect avoided would be informative.

The present results are consistent with previous findings of holistic processing of words in the context of more reading-specific contexts. They also are in line with the conception of the visual word form area as a region that subserves expert parallel processing of letters [28]. According to this dual stream model, native speakers would use this ventral route for real words and the dorsal route for pseudowords. Individuals with less expertise may rely more on the dorsal stream, or on more posterior regions of the ventral stream.

Our study represents one of the few attempts to bridge the literature on holistic processing between words and other objects of expertise by using a common paradigm (see also Farah et al. [17], and Hsiao & Cottrell [37]). The composite paradigm captures one type or aspect of holistic processing: the obligatory attention to multiple parts. Although there are other ways to assess holistic processing [11], [54], [55], [56], this paradigm has been used for many other object categories [3], [4], [5], [6], [7], and thus is an ideal method for identifying general principles of neural plasticity in object perception. Previous studies on other object categories have emphasized the need for subordinate-level discrimination for development of holistic processing [7]. It is not clear whether this principle also applies to expert word recognition, which at some level, at least, is thought to involve coarser, basic-level processing that is more part-based [7], [32], [33]. Of course, a limitation of our study is that we did not test the same participants on both words and a category involving subordinate-level expertise (typically faces) and compare their holistic processing effects. And even if the same paradigm shows similar magnitudes of holistic processing for two categories, one cannot conclude that the effects are of a similar nature. Future studies should therefore compare not only the magnitudes of but also the neural loci of and factors modulating holistic processing for different object categories.

## Supporting Information

Table S1 **Word stimuli in Experiment 1.**
(DOC)

Table S2 **Word stimuli in Experiment 2.**
(DOC)
