# Supplementary material for: Holistic Processing of Words Modulated by Reading Experience
Source: PLoS One. 2011 Jun 16;6(6):e20753. doi: 10.1371/journal.pone.0020753 (PMC3116835; doi:10.1371/journal.pone.0020753)
Supplement: Table S1 — Word stimuli in Experiment 1. (DOC) [file pone.0020753.s001.doc]

*TABLE S1*.

| Set | Word | | | |
| --- | --- | --- | --- | --- |
| 1 | crop | crew | stop | stew |
| 2 | brim | brow | slim | slow |
| 3 | clam | clip | gram | grip |
| 4 | tray | trot | play | plot |
| 5 | chat | chin | spat | spin |
| 6 | calm | cash | film | fish |
| 7 | park | past | cork | cost |
| 8 | folk | form | walk | warm |
| 9 | kick | kind | lack | land |
| 10 | sold | song | wild | wing |
