# Supplementary material for: Holistic Processing of Words Modulated by Reading Experience
Source: PLoS One. 2011 Jun 16;6(6):e20753. doi: 10.1371/journal.pone.0020753 (PMC3116835; doi:10.1371/journal.pone.0020753)
Supplement: Table S2 — Word stimuli in Experiment 2. (DOC) [file pone.0020753.s002.doc]

*TABLE S2*.

| Type | Set | Word | Kucera-Francis written frequency (per million) |
| --- | --- | --- | --- |
| High-frequency words | 1 | walk | 100 |
|  |  | warm | 67 |
|  |  | folk | 370 |
|  |  | form | 34 |
|  | 2 | belt | 29 |
|  |  | bend | 24 |
|  |  | salt | 46 |
|  |  | sand | 28 |
|  | 3 | cast | 502 |
|  |  | camp | 75 |
|  |  | just | 872 |
|  |  | jump | 24 |
|  | 4 | plan | 205 |
|  |  | plus | 72 |
|  |  | than | 1789 |
|  |  | thus | 312 |
| Low-frequency words | 5 | chat | 5 |
|  |  | chop | 3 |
|  |  | slat | 2 |
|  |  | slop | 2 |
|  | 6 | sect | 2 |
|  |  | sewn | 1 |
|  |  | pact | 5 |
|  |  | pawn | 2 |
|  | 7 | bump | 5 |
|  |  | bunt | 3 |
|  |  | ramp | 2 |
|  |  | rant | 1 |
|  | 8 | hilt | 5 |
|  |  | hind | 6 |
|  |  | melt | 4 |
|  |  | mend | 2 |
| Pseudowords | 9 | nimp | -- |
|  |  | nirk | -- |
|  |  | bomp | -- |
|  |  | bork | -- |
|  | 10 | dren | -- |
|  |  | druv | -- |
|  |  | flen | -- |
|  |  | fluy | -- |
|  | 11 | hurs | -- |
|  |  | huft | -- |
|  |  | wirs | -- |
|  |  | wift | -- |
|  | 12 | kalt | -- |
|  |  | kand | -- |
|  |  | jelt | -- |
|  |  | jend | -- |
